# Dataset on the evidence of bee products processing: A functional definition of a specialized type of macro-lithic tool

**DOI:** 10.1016/j.dib.2017.08.044

**Published:** 2017-09-09

**Authors:** Mireia Ache, Selina Delgado-Raack, Elena Molina, Roberto Risch, Antoni Rosell-Melé

**Affiliations:** a ASOME (Grup de Recerca de Arqueoecologia Social Mediterrània), Department of Prehistory, Edifici B, Universitat Autònoma de Barcelona, 08193 Bellaterra, Spain; b Institute of Environmental Science & Technology, Universitat Autònoma de Barcelona, 08193 Bellaterra, Spain; c Institució Catalana de Recerca i Estudis Avançats (ICREA), 08010 Barcelona, Catalonia, Spain

## Abstract

The database includes spatial, chronological and technological information about the analyzed tools in the article entitled “Evidence of bee products processing: a functional definition of a specialized type of macro-lithic tool” (Ache et al., 2017 [1]). The technological information refers to the tool type, its rock type, weight, state of preservation, morphology, metrical data and functional features. We also provide an index of acronyms to properly understand the dataset published here.

**Specifications Table**

**Table:** 

Subject area	*Prehistory*
More specific subject area	*Functional interpretation of an specific type of lithic tool*
Type of data	*Table*
How data was acquired	*Description of technological features of the lithic tools according to a standardized procedure* ([3]*, pp. 35–48)*
Data format	*Raw*
Experimental factors	*Archaeological stone tools were cleaned with water in order to remove sediment attached to the surface and make it visible for use-wear analysis*
Experimental features	*The database doesn’t include information about experimental features, but about the technological description of the analyzed archaeological tools*
Data source location	*See location of sites in* Ache et al. [1]*, Fig. 1*
Data accessibility	*The data are available with this article*

**Value of the data**

• The data define a specific macrolithic tool type (ALS-STA) with distinctive technological features on the basis of its geology, morphology, metrical and functional characteristics.
• Information exposed in the database offers a tracer of the specific tool type ALS-STA which helps archaeologists to recognize and define it.
• These data allow comparative studies with macrolithic tools of other areas.

## 1 Experimental design, materials and methods

Lithic tools were registered following a standardized recording of their geological, morphological, metrical and functional variables according to Risch [3], pp. 35–48 and Delgado-Raack [2], pp. 187–199. The acronyms used in Table 1 and their meaning are the following ones:

• **No. Inv L-**. Inventory number or individual number of the archaeological artefact.
• **YACIM**. Name of the archaeological site.
• **No. Sondeo**. Trench.
• **ZON**. Area within the archaeological site.
• **Fase**. Cultural phase.
• **Horiz**. Cultural horizon.
• **ITEM**. Tool name: Abrader (ALS), abrader-percussor (APE).
• **TIPO**. Tool's specific type: Cylindrical (STA), with groove (CRN).
• **MATERIA**. Rock type: Calcarenite (ACA), sandstone (ARE), Buntsandstein (BUN), limestone (CAL), quartzite (CCT), micaceous schist (ESM), quartzitic schist (ESQ), gneiss (GNE), metapsammite (MPS), slate (PZA).
• **PESO**. Weight in g. * indicates that the artefact is not completely preserved.
• **CONS**. Preservation of the artefact taking into account that each artefact is divided into three parts: complete artefact (ENT), top and medial fragment (FSM), medial and bottom fragment (FMI), top fragment (FGS), medial fragment (FGM).
• **No. FR**. Number of fragments.
• **F**. Morphology of the six surfaces into which the artefact is divided: straight (RT), concave (CV), convex (CX). The morphology is recorded for both the longitudinal and the transversal axis and for each of the six surfaces, that is to say, obverse (F.A.1, F.A.2), reverse (F.R.1, F.R.2), top (F.S.1, F.S.2), bottom (F.I.1, F.I.2), right (F.D.1, F.D.2) and left (F.X.1, F.X.2).
• **L.MAX**. Maximum length measured in mm. * indicates that the artefact is not completely preserved.
• **A.MAX.; A.MIN**. Maximum and minimum width measured in mm. * indicates that the artefact is not completely preserved.
• **G.MAX.; G.MIN**. Maximum and minimum thickness measured in mm. * indicates that the artefact is not completely preserved.
• **UTI.A.; UTI.R.; UTI.S.; UTI.I.; UTI.D.; UTI.X**. Macroscopic use-wear traces observed on each of the six surfaces into which the artefact is divided: naturally smooth surface (LI), naturally irregular surface (IR), abrasion due to the use of the surface in frictional tasks (AL), abrasion/smoothing due to the preparation of the surface (PU), groove (RA), pits due to the use of the surface in percussion tasks (GA), pits and breaks due to the use of the surface in percussion tasks (GO), surface prepared through chipping (TR), broken surface (RO), burned surface (TE).
• **MED.A.1; MED.A.2; MED.R.1; MED.R.2; MED.S.1; MED.S.2; MED.I.1; MED.I.2; MED.D.1; MED.D.2; MED.X.1; MED.X.2**. Dimensions of the macroscopic use-wear traces observed on each of the six surfaces into which the artefact is divided (obverse, reverse, top, bottom, right and left) in its longitudinal (1) and its transversal (2) axis. * indicates that the artefact is not completely preserved.
• **UTI.ESP**. Special macroscopic features, mainly burned surfaces, which appear in these cases as an effect of use (TE).
• **SIT.UTI.ESP**. Location of UTI.ESP., according to the six surfaces (obverse, reverse, top, bottom, right, left) into which the artefact is divided.
• **L.ESP.; A.ESP.; P.ESP**. Length, width and thickness of UTI.ESP, measured in mm.

**Table 1:** Main technological features of the cylindrical abraders recovered in south-east Iberian Bronze Age sites.

**No. Inv L-**	**YACIM**	**No. Sondeo**	**ZON**	**Fase**	**Horiz.**	**ITEM**	**TIPO**	**MATERIA**
**AR-L-009**	El Argar	3				ALS	STA	ESM
**AR-L-010**	El Argar	3				ALS	STA	CCT
**AR-L-015**	El Argar	1				APE	STA	ESQ
**AR-L-017**	El Argar	1				ALS	STA	ESM
**AR-L-044**	El Argar	3				ALS	STA	ESM
**AR-L-043**	El Argar	Bibliogr				ALS	STA	–
**AR-L-064**	El Argar	Bibliogr				ALS?	STA	–
**AR-L-065**	El Argar	Bibliogr				ALS?	STA	–
**AR-L-065**	El Argar	Bibliogr				ALS?	STA	–
**AR-L-158**	El Argar	Bibliogr				ALS?	STA	PZA
**AR-L-159**	El Argar	Bibliogr				ALS?	STA	–
**AR-L-160**	El Argar	Bibliogr				ALS?	STA	PZA
**BA-A8-9.2**	La Bastida		6			ALS	STA	ESQ
**BA-E00-113.2**	La Bastida		4			ALS	STA	MPS
**BA-E00-166.4**	La Bastida					ALS	STA	ESQ
**BA-E00-24.8**	La Bastida		1			ALS	STA	CAL
**BA-E16-27.5**	La Bastida		3	3B		ALS	STA	CAL
**BA-E16-5.17**	La Bastida		3	3B		ALS	STA?	BUN
**BA-E7-7.11**	La Bastida		1			ALS	STA	MPS
**BA-H1-8.4**	La Bastida		1	3B		ALS	STA	CCT
**BA-H20-26**	La Bastida		1	3A		ALS	STA	ESQ
**BA-H2-170**	La Bastida		1	3B		ALS	STA	ESQ
**BA-H3-154**	La Bastida		1	3B		ALS	STA	ESQ
**BA-H36-232.1**	La Bastida		0	1		ALS	STA	CAL
**BA-H36-232.2**	La Bastida		0	1		ALS	STA	ARE
**BA-H36-691.1**	La Bastida		0	1		ALS	STA?	ESM
**BA-H37-24.1**	La Bastida		0	3B		ALS	STA	ESQ
**BA-H3-75**	La Bastida		1	3B		ALS	STA	ESQ
**BA-H52-70**	La Bastida		0	3A		ALS	STA	ESQ
**BA-H53-4.6**	La Bastida		7	3B		ALS	STA	ESQ
**BA-H61-38**	La Bastida		3	3B		ALS	STA	ESQ
**BA-H64-5**	La Bastida		7	2		ALS	STA	ESQ
**BA-I2-46.2**	La Bastida		1	2		ALS	STA	CAL
**BA-I2-9.22**	La Bastida		1	2		ALS	STA?	ARE
**TO-5**	La Bastida	Bibliogr				ALS	STA	PZA?
**TO-6**	La Bastida	Bibliogr				ALS	STA	PZA?
**CN-1**	Cabezo Negro	Bibliogr				ALS?	STA	–
**CR-16**	Cabezo Redondo	Bibliogr				ALS?	STA?	PZA?
**CR-17**	Cabezo Redondo	Bibliogr				ALS?	STA?	ARE
**FA-L-0378**	Fuente Álamo	23–24	O/SO	12B	III/IV	ALS	STA	PZA
**FA-L-0517**	Fuente Álamo	7	O/NO	7	I/II	ALS	STA	MPS
**FA-L-0536**	Fuente Álamo			20	I–VII	ALS	STA	ESQ
**FA-L-0541**	Fuente Álamo	5	O/SO	14–17		ALS	STA	ESQ
**FA-L-0565**	Fuente Álamo	3	O/SO	16/17	V	APE	STA	ESQ
**FA-L-0587**	Fuente Álamo	35	O/NO			ALS	STA	PZA

**No. Inv L-**	**PESO**	**CONS.**	**No. FR.**	**F.A.1**	**F.A.2**	**F.R.1**	**F.R.2**
**AR-L-009**	330	ENT	1	RT	CX	CX	RT
**AR-L-010**	260	ENT	1	RT	RT	RT	RT
**AR-L-015**	320	ENT	1	RT	RT	RT	RT
**AR-L-017**	210	ENT	1	RT	CX	CX	RT
**AR-L-044**	265	ENT	1	RT	CX	RT	RT
**AR-L-043**							
**AR-L-064**							
**AR-L-065**							
**AR-L-065**							
**AR-L-158**							
**AR-L-159**							
**AR-L-160**							
**BA-A8-9.2**	281	ENT	1	RT	CX	CX	CX
**BA-E00-113.2**	102	ENT	1	CX	CX	RT	CX
**BA-E00-166.4**	215	ENT	1	RT	CX	RT	RT
**BA-E00-24.8**	164	ENT	1	RT	CX	RT	CX
**BA-E16-27.5**	226	ENT	1	RT	RT	CX	CX
**BA-E16-5.17**	471	ENT	1	CX	CV	IR	RT
**BA-E7-7.11**	140	ENT	1	CX	CX	RT	CX
**BA-H1-8.4**	297	ENT	1	CX	CX	CX	CX
**BA-H20-26**	559	ENT	2	IR	RT	RT	RT
**BA-H2-170**	289	ENT	1	CX	CX	CX	CX
**BA-H3-154**	200	ENT		CX	CX	CX	CX
**BA-H36-232.1**	169	ENT	1	CX	CX	CV	CX
**BA-H36-232.2**	165*	FMI	1	CX	CX	RT	CX
**BA-H36-691.1**	337	ENT	1	CV	CX	CV	RT
**BA-H37-24.1**	147	ENT	1	CX	CX	CV	CV
**BA-H3-75**	775	ENT	2	RT	CX	CV	CX
**BA-H52-70**	226*	FMI	1	RT	RT	CX	CX
**BA-H53-4.6**	141*	FSM	1	IR	CX	IR	IR
**BA-H61-38**	224	ENT	1	CX	CX	IR	CX
**BA-H64-5**	90*	FSM	1	RT	CX	CX	CX
**BA-I2-46.2**	215	ENT	1	CX	CX	CX	CX
**BA-I2-9.22**	26*	FGS					
**TO-5**							
**TO-6**							
**CN-1**							
**CR-16**							
**CR-17**							
**FA-L-0378**	315	ENT	1	RT	CX	CX	RT
**FA-L-0517**	185	ENT	1	RT	CX	CX	RT
**FA-L-0536**	600	ENT	1	RT	CX	CX	RT
**FA-L-0541**	300*	FSM	1	CX	RT	RT	RT
**FA-L-0565**	655*	FSM	1	RT	CX	CX	RT
**FA-L-0587**	175	ENT	1	RT	CX	CX	RT

**No. Inv L-**	**F.S.1**	**F.S.2**	**F.I.1**	**F.I.2**	**F.D.1**	**F.D.2**
**AR-L-009**	CX	CX	CX	RT	RT	CX
**AR-L-010**	CX	CX	CX	RT	RT	CX
**AR-L-015**	CX	CX	CX	RT	RT	CX
**AR-L-017**	RT	RT	CX	RT	RT	CX
**AR-L-044**	CX	CX	CX	RT	RT	CX
**AR-L-043**						
**AR-L-064**						
**AR-L-065**						
**AR-L-065**						
**AR-L-158**						
**AR-L-159**						
**AR-L-160**						
**BA-A8-9.2**	CX	CX	CX	CX	CX	CX
**BA-E00-113.2**	CX	CX	CX	CX	RT	CX
**BA-E00-166.4**	CX	CX	CX	CX	RT	CX
**BA-E00-24.8**	IR	CX	CX	CX	CX	CX
**BA-E16-27.5**	CX	CX	CX	CX	CX	CX
**BA-E16-5.17**	CX	CX	CX	CX	IR	CX
**BA-E7-7.11**	CX	CX	IR	IR	RT	RT
**BA-H1-8.4**	CX	CX	CX	CX	CX	CX
**BA-H20-26**	CX	CX	CX	CX	IR	CX
**BA-H2-170**	CX	CX	CX	CX	CX	CX
**BA-H3-154**	CX	CX	CX	CX	CX	CX
**BA-H36-232.1**	RT	RT	CX	CX	CV	CX
**BA-H36-232.2**	RO	RO	CX	CX	CX	CX
**BA-H36-691.1**	IR	IR	CX	CX	IR	CX
**BA-H37-24.1**	CX	CX	CX	CX	IR	CX
**BA-H3-75**	CX	CX	CX	CX	RT	CX
**BA-H52-70**	RO	RO	CX	AG	CX	CX
**BA-H53-4.6**	CX	CX	RO	RO	IR	CX
**BA-H61-38**	CX	CX	CX	CX	CX	CX
**BA-H64-5**	CX	CX	RO	RO	RT	CX
**BA-I2-46.2**	CX	CX	CX	CX	CX	CX
**BA-I2-9.22**						
**TO-5**						
**TO-6**						
**CN-1**						
**CR-16**	RO	RO				
**CR-17**	RT	RT				
**FA-L-0378**	CX	CX	CX	CX	RT	CX
**FA-L-0517**	CX	CX	CX	CX	IR	CX
**FA-L-0536**	CX	CX	CX	CX	RT	CX
**FA-L-0541**	CX	CX	RO	RO	RT	CX
**FA-L-0565**	CX	CX	RO	CX	RT	CX
**FA-L-0587**	CX	CX	CX	CX	RT	CX

**No. Inv L-**	**F.X.1**	**F.X.2**	**L.MAX.**	**A.MAX.**	**A.MIN.**
**AR-L-009**	RT	CX	183	41	
**AR-L-010**	RT	RT	158	39	
**AR-L-015**	RT	CX	160	51	
**AR-L-017**	RT	CX	131	50	
**AR-L-044**	RT	CX	149	41	
**AR-L-043**			172		
**AR-L-064**			160	48	
**AR-L-065**			100	18	
**AR-L-065**			134	28	
**AR-L-158**			200	26	
**AR-L-159**			83	12	
**AR-L-160**			104	23	
**BA-A8-9.2**	CX	CX	153	41	
**BA-E00-113.2**	RT	CX	101	33	
**BA-E00-166.4**	RT	CX	160	33	
**BA-E00-24.8**	RT	CX	77	36	
**BA-E16-27.5**	CV	CX	145	42	
**BA-E16-5.17**	IR	CX	173	89	
**BA-E7-7.11**	CX	CX	123	27	
**BA-H1-8.4**	CX	CX	119	57	
**BA-H20-26**	RT	CX	209	40	
**BA-H2-170**	CX	CX	122	44	
**BA-H3-154**	RT	RT	143	34	
**BA-H36-232.1**	CX	CX	114	36	
**BA-H36-232.2**	RT	CX	105*	42*	
**BA-H36-691.1**	IR	CX	129	71	
**BA-H37-24.1**	IR	CX	150	32	
**BA-H3-75**	RT	CX	226	49	
**BA-H52-70**	RT	CX	105*	62*	49
**BA-H53-4.6**	CV	CX	112*	37*	
**BA-H61-38**	CX	CX	199	33	
**BA-H64-5**	CX	CX	92*	38*	
**BA-I2-46.2**	CV	CX	101	39	
**BA-I2-9.22**					
**TO-5**			84	30	
**TO-6**			92	24	
**CN-1**				43	
**CR-16**				14	
**CR-17**			155	17	
**FA-L-0378**	RT	CX	170	44	
**FA-L-0517**	RT	CX	132	30	
**FA-L-0536**	RT	CX	211	57	
**FA-L-0541**	CX	CX	193*	51	
**FA-L-0565**	CX	CX	183	61	
**FA-L-0587**	CX	CX	155	39	

**No. Inv L-**	**G.MAX.**	**G.MIN.**	**UTI.A.**	**UTI.R.**	**UTI.S.**
**AR-L-009**	26		LI	PU	AL
**AR-L-010**	30		LI	AL	AL
**AR-L-015**	20		LI	LI	AL
**AR-L-017**	22		LI	LI	AL
**AR-L-044**	27		LI	LI	AL
**AR-L-043**	24				AL
**AR-L-064**					
**AR-L-065**					
**AR-L-065**					
**AR-L-158**	17				
**AR-L-159**	12				
**AR-L-160**	12				
**BA-A8-9.2**	26		LI	LI	AL/TE
**BA-E00-113.2**	23		LI	LI	AL
**BA-E00-166.4**	22		LI	–	AL
**BA-E00-24.8**	35		LI	LI	TR
**BA-E16-27.5**	27		AL	LI	AL/TE
**BA-E16-5.17**	28		AL	LI	AL/TE
**BA-E7-7.11**	27		LI	LI	AL/TE
**BA-H1-8.4**	28		LI	LI	AL/TE
**BA-H20-26**	35		LI	LI	AL/TE
**BA-H2-170**	40		LI	LI	GA/AL/TE
**BA-H3-154**	27		LI	LI	GA
**BA-H36-232.1**	27		AL	AL	AL
**BA-H36-232.2**	28*		AL	AL	RO
**BA-H36-691.1**	30		AL	LI	IR
**BA-H37-24.1**	19				AL/TE
**BA-H3-75**	34		AL	AL	
**BA-H52-70**	25*		AL	–	RO
**BA-H53-4.6**	22*		LI	LI	AL/TE?
**BA-H61-38**	22		LI	LI	–
**BA-H64-5**	22*		LI	–	AL?
**BA-I2-46.2**	36		LI	LI	GA/AL/TE
**BA-I2-9.22**					AL
**TO-5**	14		LI		AL
**TO-6**	20		LI		AL
**CN-1**	21		AL?		LI?
**CR-16**	6		AL?	PU	PU
**CR-17**	15		AL	AL?	PU
**FA-L-0378**	23		AL/LI?	LI	AL
**FA-L-0517**	22		LI	LI	AL
**FA-L-0536**	33		AL	PU	AL
**FA-L-0541**	25		AL	IR	AL
**FA-L-0565**	42		AL	LI	AL
**FA-L-0587**	23		LI	LI	AL

**No. Inv L-**	**UTI.I.**	**UTI.D.**	**UTI.X.**	**MED.A.1**	**MED.A.2**
**AR-L-009**	AL	AL	PU		
**AR-L-010**	AL	PU	PU		
**AR-L-015**	LI	LI	LI		
**AR-L-017**	GO	LI	LI		
**AR-L-044**	LI	LI	LI		
**AR-L-043**					
**AR-L-064**					
**AR-L-065**					
**AR-L-065**					
**AR-L-158**					
**AR-L-159**					
**AR-L-160**					
**BA-A8-9.2**	AL	LI	LI		
**BA-E00-113.2**	GA/AL	LI	RO		
**BA-E00-166.4**	AL/TE	LI	LI		
**BA-E00-24.8**	AL/TE	LI	LI	33	30
**BA-E16-27.5**	AL/TE	LI	LI	113	34
**BA-E16-5.17**	GA?	LI	LI	152	75
**BA-E7-7.11**	IR	LI	LI		
**BA-H1-8.4**	AL/TE	LI	LI		
**BA-H20-26**	AL/TE	LI	LI		
**BA-H2-170**	GA/TE	LI	AL		
**BA-H3-154**	GO/AL	LI	LI		
**BA-H36-232.1**	AL/TE	AL	AL	72	36
**BA-H36-232.2**	AL/TE	AL	AL	48*	39*
**BA-H36-691.1**	AL/TE	LI	LI	98	64
**BA-H37-24.1**	AL/TE				
**BA-H3-75**	AL/TE	LI	LI		
**BA-H52-70**	AL/TE			103*	57*
**BA-H53-4.6**	RO	LI	LI		
**BA-H61-38**	AL	LI	LI		
**BA-H64-5**	RO	LI	LI		
**BA-I2-46.2**	LI	LI	LI		
**BA-I2-9.22**					
**TO-5**					
**TO-6**					
**CN-1**					
**CR-16**					
**CR-17**					
**FA-L-0378**	LI	LI	LI	72	34
**FA-L-0517**	AL	LI	LI		
**FA-L-0536**	AL	PU	PU		
**FA-L-0541**	RO	PU-LI?	PU-LI?		
**FA-L-0565**	GO	LI	GO		
**FA-L-0587**	LI	AL	LI		

**No. Inv L-**	**MED.R.1**	**MED.R.2**	**MED.S.1**	**MED.S.2**	**MED.I.1**
**AR-L-009**			35	15	28
**AR-L-010**	117	25			
**AR-L-015**					35
**AR-L-017**					
**AR-L-044**			32	13	23
**AR-L-043**					
**AR-L-064**					
**AR-L-065**					
**AR-L-065**					
**AR-L-158**					
**AR-L-159**					
**AR-L-160**					
**BA-A8-9.2**			46	23	47
**BA-E00-113.2**			14	12	22/8(GA)
**BA-E00-166.4**			32*	22	60
**BA-E00-24.8**					
**BA-E16-27.5**			51/51	40/40	37/60
**BA-E16-5.17**			45	25	
**BA-E7-7.11**			29	29	
**BA-H1-8.4**			50	22	49
**BA-H20-26**			52	44	46
**BA-H2-170**			28	18	51
**BA-H3-154**			11	7	20/17(GO)
**BA-H36-232.1**	67	35	36	27	61
**BA-H36-232.2**	65*	38*			57
**BA-H36-691.1**					32
**BA-H37-24.1**			59	29	19
**BA-H3-75**					120
**BA-H52-70**					54
**BA-H53-4.6**			37	22	
**BA-H61-38**			81	25	
**BA-H64-5**					
**BA-I2-46.2**			14/12(GA)	35/27(AL/TE)	
**BA-I2-9.22**					
**TO-5**					
**TO-6**					
**CN-1**					
**CR-16**					
**CR-17**					
**FA-L-0378**					
**FA-L-0517**			28	23	27
**FA-L-0536**			49	47	43
**FA-L-0541**					
**FA-L-0565**					
**FA-L-0587**			16	11	

**No. Inv L-**	**MED.I.2**	**MED.D.1**	**MED.D.2**	**MED.X.1**	**MED.X.2**
**AR-L-009**	9				
**AR-L-010**					
**AR-L-015**	17				
**AR-L-017**					
**AR-L-044**	18				
**AR-L-043**					
**AR-L-064**					
**AR-L-065**					
**AR-L-065**					
**AR-L-158**					
**AR-L-159**					
**AR-L-160**					
**BA-A8-9.2**	25				
**BA-E00-113.2**	31/27(AL)				
**BA-E00-166.4**	25*				
**BA-E00-24.8**					
**BA-E16-27.5**	42/42				
**BA-E16-5.17**					
**BA-E7-7.11**					
**BA-H1-8.4**	36				
**BA-H20-26**	34				
**BA-H2-170**	11				
**BA-H3-154**	32/31(AL)				
**BA-H36-232.1**	22	109	57	10	65
**BA-H36-232.2**	32	38*	18	38*	22
**BA-H36-691.1**	27				
**BA-H37-24.1**	20				
**BA-H3-75**	49				
**BA-H52-70**	49				
**BA-H53-4.6**					
**BA-H61-38**					
**BA-H64-5**					
**BA-I2-46.2**					
**BA-I2-9.22**					
**TO-5**					
**TO-6**					
**CN-1**					
**CR-16**					
**CR-17**					
**FA-L-0378**					
**FA-L-0517**	17				
**FA-L-0536**	50				
**FA-L-0541**					
**FA-L-0565**					
**FA-L-0587**		60	11		

**No. Inv L-**	**UTI.ESP.**	**SIT.UTI.ESP.**	**L.ESP.**	**A.ESP.**	**P.ESP.**
**AR-L-009**	TE	S	38	18	22
**AR-L-010**	TE	S	30	30	35
**AR-L-015**					
**AR-L-017**					
**AR-L-044**					
**AR-L-043**					
**AR-L-064**					
**AR-L-065**					
**AR-L-065**					
**AR-L-158**					
**AR-L-159**					
**AR-L-160**					
**BA-A8-9.2**	TE				
**BA-E00-113.2**	TE				
**BA-E00-166.4**	TE				
**BA-E00-24.8**	TE				
**BA-E16-27.5**	TE				
**BA-E16-5.17**	TE				
**BA-E7-7.11**	TE				
**BA-H1-8.4**	TE				
**BA-H20-26**	TE				
**BA-H2-170**	TE	S/I	52/42	25/23	
**BA-H3-154**					
**BA-H36-232.1**	TE	I	83/68	27/23	
**BA-H36-232.2**	TE	I	58/52	21/25	
**BA-H36-691.1**					
**BA-H37-24.1**	TE	S/I	59/19	30/19	
**BA-H3-75**	TE				
**BA-H52-70**	TE	98	49		
**BA-H53-4.6**	TE?				
**BA-H61-38**					
**BA-H64-5**	–				
**BA-I2-46.2**	TE				
**BA-I2-9.22**	TE				
**TO-5**	TE	S;I			
**TO-6**	TE	S;I			
**CN-1**					
**CR-16**					
**CR-17**	TE	TE			
**FA-L-0378**	TE	A	5	38	18
**FA-L-0517**	TE	S; I			
**FA-L-0536**	TE	S; I			
**FA-L-0541**	TE	S			
**FA-L-0565**	TE	S	47	33	
**FA-L-0587**					

**No. Inv L-**	**YACIM**	**No. Sondeo**	**ZON**	**Fase**	**Horiz.**	**ITEM**	**TIPO**	**MATERIA**
**FA-L-0599**	Fuente Álamo	3	O/SO	16–18		ALS	STA	PZA
**FA-L-0600**	Fuente Álamo	34	O/NW	16/17	V	ALS	STA	PZA
**FA-L-0603**	Fuente Álamo	4/27 Steg	O/SW	19		ALS	STA	PZA
**FA-L-0613**	Fuente Álamo	32	W/N			ALS	STA	PZA
**FA-L-0629**	Fuente Álamo	1 (0)	O/NW			ALS	STA	ESM
**FA-L-0638**	Fuente Álamo	7 (6/7)	O/NO	11B	III/IV	ALS	STA	PZA
**FA-L-0644**	Fuente Álamo	12	O/NO	5C	I/II	ALS	STA	PZA
**FA-L-0647**	Fuente Álamo	3	O/SO	15	III/IV	ALS	STA	PZA
**FA-L-0650**	Fuente Álamo	4	O/SW	16/17	V	ALS	STA	PZA
**FA-L-0658**	Fuente Álamo	6W	O/NW	16/17	V	ALS	STA	PZA
**FA-L-0660**	Fuente Álamo	6	O/NO	8B/9	I/II	ALS	STA	ESQ
**FA-L-0670**	Fuente Álamo	2/6	O/NO	15 B	III/IV	ALS	STA	ESM
**FA-L-0672**	Fuente Álamo	3/5	O/SO	16/17	V	ALS	STA	PZA
**FA-L-0678**	Fuente Álamo	4	O/SW	18	III/IV	ALS	STA	PZA
**FA-L-0682**	Fuente Álamo	3	O/SO	14	III/IV	ALS	STA	PZA
**FA-L-0684**	Fuente Álamo	20	N			ALS	STA	PZA
**FA-L-0756**	Fuente Álamo	6W	O/NW	11	III/IV	APE	STA	ESQ
**FA-L-0766**	Fuente Álamo	23/24	O/SO	7–9	I/II	ALS	STA	ESQ
**FA-L-0771**	Fuente Álamo	Gr. 98				ALS	STA	PZA
**FA-L-0785**	Fuente Álamo	19	W/S			ALS	STA	ESM
**FA-L-0797**	Fuente Álamo	29	O/SW	20	I–VII	ALS	STA	PZA
**FA-L-0954**	Fuente Álamo	35W	O/NW	16/17	V	ALS	STA	ESQ
**FA-L-0955**	Fuente Álamo	20/35 Steg	O/NO			ALS	STA	PZA
**FA-L-0958**	Fuente Álamo	20/35 Steg	O/NO			ALS	STA	ESQ
**FA-L-0972**	Fuente Álamo	35W	O/NW	17	V	ALS	STA	PZA
**FA-L-0983**	Fuente Álamo	35W	O/NW	17	V	ALS	STA	PZA
**FA-L-0995**	Fuente Álamo	39	S			ALS	STA	PZA
**FA-L-0997**	Fuente Álamo	35W	O/NW	20	I–VII	ALS	STA	ESQ
**FA-L-1006**	Fuente Álamo	35 W-G.105	O/NW	13b-15	III/IV	ALS	STA	PZA
**FA-L-1007**	Fuente Álamo	35W	O/NW	17	V	ALS	STA	PZA
**FA-L-1008**	Fuente Álamo	35W	O/NW	17	V	ALS	STA	PZA
**FA-L-1010**	Fuente Álamo	41	S		C	ALS	STA	ESM
**FA-L-1011**	Fuente Álamo	19/30 Steg	W/S			ALS	STA	PZA
**FA-L-1012**	Fuente Álamo	19/30 Steg	W/S			ALS	STA	PZA
**FA-L-1032**	Fuente Álamo	35W	O/NW	16/17	V	ALS	STA	PZA
**FA-L-1040**	Fuente Álamo	41	S		A	APE	STA	ESQ
**FA-L-1299**	Fuente Álamo					ALS	STA	PZA
**FA-L-781B**	Fuente Álamo	29	O/SW	20	I–VII	APE	STA	PZA
**FA-L-787B**	Fuente Álamo	41	S	20	I–VII	ALS	STA	ESQ
**FV-6**	Fuente Vermeja	Bibliogr				ALS	STA?	–
**G-1995ZC-L-506**	Gatas	109/T4		3IV		ALS	STA	ESQ
**G-1995-ZC-L-589**	Gatas	ZC	212B3	IV		ALS	STA	ESM
**G-1995ZC-L-596**	Gatas		212A1	IV		ALS	STA	ESM
**G-2001MS-L-048**	Gatas		5	III–IV		ALS	STA	PZA
**G-2001MS-L-079**	Gatas		10	III–IV		ALS	STA?	PZA

**No. Inv L-**	**PESO**	**CONS.**	**No. FR.**	**F.A.1**	**F.A.2**	**F.R.1**	**F.R.2**
**FA-L-0599**	180	ENT	1	IR	CX	RT	RT
**FA-L-0600**	105	ENT	1	RT	RT	CX	RT
**FA-L-0603**	135*	FSM	1	RT	RT	CX	RT
**FA-L-0613**	325*	FSM	1	CV	RT	CX	CX
**FA-L-0629**	225	ENT	1	RT	CX	CX	RT
**FA-L-0638**	75	ENT	1	RT	CX	CX	RT
**FA-L-0644**	350	ENT	1	RT	CX	CX	RT
**FA-L-0647**	385*	FSM	1	CV	CX	CX	CX
**FA-L-0650**	375	ENT	1	RT	CX	CX	RT
**FA-L-0658**	70*	FGS	1	RT	CX	RT	RT
**FA-L-0660**	70*	FGS	1	RT	RT	CX	RT
**FA-L-0670**	225*	FGS	1	RT	CX	CX	RT
**FA-L-0672**	450	ENT	1	RT	RT	CX	RT
**FA-L-0678**	185*	FGT	1	RT	RT	CX	RO
**FA-L-0682**	110*	FGS	1	RT	RT	CX	RT
**FA-L-0684**	100*	FSM	1	RT	RT	CX	RT
**FA-L-0756**	320	ENT	1	RT	CX	CX	RT
**FA-L-0766**	170	ENT	1	RT	CX	CX	RT
**FA-L-0771**	60*	FSM	1	RT	CX	CX	RT
**FA-L-0785**	350	ENT	1	RT	RT	CX	CX
**FA-L-0797**	310*	FSM	1	RT	CX	CX	RT
**FA-L-0954**	185*	FGS	1	RT	CX	CX	RT
**FA-L-0955**	100*	FSM	1	RT	RT	CX	RT
**FA-L-0958**	430	ENT	1	RT	CX	CX	RT
**FA-L-0972**	195	ENT	1	RT	IR	CX	IR
**FA-L-0983**	135*	FGT	1	RT	CX	CX	RT
**FA-L-0995**	380	ENT	1	RT	CX	RT	RT
**FA-L-0997**	40*	FGS	1	RT	CX	CX	RT
**FA-L-1006**	70*	FGT	2	RT		RO	CX
**FA-L-1007**	460	ENT	1	RT	CX	CX	RT
**FA-L-1008**	160*	FSM	1	RT	RT	CX	RT
**FA-L-1010**	390*	FSM	1	RT	CX	CX	RT
**FA-L-1011**	280	ENT	1	RT	RT	CX	RT
**FA-L-1012**	360	ENT	1	RT	CX	CX	RT
**FA-L-1032**	320	ENT	1	IR	CX	CX	RT
**FA-L-1040**	450	ENT	1	CX	CX	CX	RT
**FA-L-1299**	420	ENT	1	RT	CX	RT	CX
**FA-L-781B**	150	ENT	1	RT	CX	CX	RT
**FA-L-787B**	70*	FGS	1	RT	RT	CX	RT
**FV-6**							
**G-1995ZC-L-506**	744	ENT	1	CX	CX	RT	RT
**G-1995-ZC-L-589**	196	ENT	1	RT	CX	RT	RT
**G-1995ZC-L-596**	220	ENT	1	RT	RT	RT	RT
**G-2001MS-L-048**	378	(ENT)	1	CX	CX	CX	CX
**G-2001MS-L-079**	62*	FGM	1	RT	CX	RT	CX

**No. Inv L-**	**F.S.1**	**F.S.2**	**F.I.1**	**F.I.2**	**F.D.1**	**F.D.2**
**FA-L-0599**	CX	RT	CX	CX	RT	AG
**FA-L-0600**	CX	CX	CX	CX	CV	CX
**FA-L-0603**	CX	CX	RO	RO	RT	CX
**FA-L-0613**	CX	AG	RO	RO	RT	CX
**FA-L-0629**	CX	AG	CX	CX	RT	CX
**FA-L-0638**	CX	CX	CX	CX	RT	CX
**FA-L-0644**	CX	CX	CX	CX	RT	CX
**FA-L-0647**	CX	CX	RO	RO	CV	CX
**FA-L-0650**	CX	CX	CX	CX	RT	CX
**FA-L-0658**	CX	CX	RO	RO	RT	CX
**FA-L-0660**	CX	CX	RO	RO	RT	CX
**FA-L-0670**	CX	CX	RO	RO	RT	CX
**FA-L-0672**	CX	CX	CX	CX	IR	CX
**FA-L-0678**	CX	CX	RO	RO	CX	CX
**FA-L-0682**	CX	CX	RO	RO	RT	CX
**FA-L-0684**	CX	CX	RO	RO	RT	CX
**FA-L-0756**	CX	AG	CX	CX	RT	CX
**FA-L-0766**	CX	CX	CX	CX	RT	CX
**FA-L-0771**	CX	CX	RO	RO	RT	CX
**FA-L-0785**	CX	CX	CX	CX	CX	CX
**FA-L-0797**	CX	CX	CX	RO	RT	CX
**FA-L-0954**	CX	CX	RO	RO	RT	CX
**FA-L-0955**	CX	CX	RO	RO	RT	CX
**FA-L-0958**	CX	CX	CX	CX	RT	CX
**FA-L-0972**	CX	CX	IR	CX	RT	CX
**FA-L-0983**	CX	CX	RO	RO	RT	RT
**FA-L-0995**	CX	CX	CX	IR	RT	CX
**FA-L-0997**	CX	CX	RO	RO	RT	CX
**FA-L-1006**	CX	CX	RO	RO	RT	CX
**FA-L-1007**	CX	CX	CX	CX	RT	CX
**FA-L-1008**	CX	CX	RO	RO	RT	CX
**FA-L-1010**	CX	CX	RO	RO	RT	CX
**FA-L-1011**	CX	CX	CX	CX	IR	CX
**FA-L-1012**	CX	CX	CX	CX	RT	CX
**FA-L-1032**	CX	CX	CX	CX	IR	CX
**FA-L-1040**	CX	CX	CX	CX	RT	CX
**FA-L-1299**	CX	IR	CX	IR	RT	RT
**FA-L-781B**	CX	CX	CX	CX	RT	CX
**FA-L-787B**	CX	CX	RO	RO	RT	CX
**FV-6**	CX	AG				
**G-1995ZC-L-506**	CX	CX	CX	CX	CX	CX
**G-1995-ZC-L-589**	CX	CX	CX	AG	RT	CX
**G-1995ZC-L-596**	CX	CX	CX	CX	RT	RT
**G-2001MS-L-048**	CX	CX	CX	CX	CV	RT
**G-2001MS-L-079**	RO	RO	RO	RO	RT	CX

**No. Inv L-**	**F.X.1**	**F.X.2**	**L.MAX.**	**A.MAX.**	**A.MIN.**
**FA-L-0599**	RT	CX	194	37	
**FA-L-0600**	CV	CX	141	29	
**FA-L-0603**	RT	CX	105*	40	
**FA-L-0613**	RT	CX	167*	40	
**FA-L-0629**	RT	CX	164	38	
**FA-L-0638**	RT	CX	170	23	
**FA-L-0644**	CX	CX	200	50	
**FA-L-0647**	CX	CX	197*	51	
**FA-L-0650**	RT	CX	233	41	
**FA-L-0658**	RT	CX	110*	28	
**FA-L-0660**	RT	CX	66*		
**FA-L-0670**	RT	CX	84*	37	
**FA-L-0672**	IR	CX	187	45	
**FA-L-0678**	RO	RO	149*	34*	
**FA-L-0682**	RT	CX	114*	34	
**FA-L-0684**	RT	CX	105	39	
**FA-L-0756**	RT	RT	179	35	
**FA-L-0766**	RT	CX	186	28	
**FA-L-0771**	RT	CX	86*	29	
**FA-L-0785**	CX	CX	135	51	
**FA-L-0797**	RT	CX	167*	48	
**FA-L-0954**	RT	CX	75*	34	
**FA-L-0955**	RT	CX	94*	35	
**FA-L-0958**	RT	CX	207	43	
**FA-L-0972**	RT	CX	143	39	
**FA-L-0983**	RO	RO	118*	33	
**FA-L-0995**	RT	CX	194	54	
**FA-L-0997**	RT	CX	64*	23	
**FA-L-1006**	RO	RO	125*	12*	
**FA-L-1007**	RT	CX	222	45	
**FA-L-1008**	RT	CX	113*	43	
**FA-L-1010**	RT	CX	131*	52	
**FA-L-1011**	RT	CX	181	42	
**FA-L-1012**	IR	CX	192	50	
**FA-L-1032**	RT	CX	194	46	
**FA-L-1040**	RT	CX	150	57	
**FA-L-1299**	CX	RT	173	51	
**FA-L-781B**	RT	CX	124	32	
**FA-L-787B**	RT	RT	59*	24	
**FV-6**			190	35	
**G-1995ZC-L-506**	RT	CX	203	60	
**G-1995-ZC-L-589**	RT	CX	148	35	
**G-1995ZC-L-596**	RT	CX	164	36	
**G-2001MS-L-048**	CX	CX	165		42
**G-2001MS-L-079**	RT	CX	130*	21	

**No. Inv L-**	**G.MAX.**	**G.MIN.**	**UTI.A.**	**UTI.R.**	**UTI.S.**
**FA-L-0599**	23		LI	LI	AL
**FA-L-0600**	16		AL	LI	AL
**FA-L-0603**	22		AL	LI	AL
**FA-L-0613**	29		PU	PU/AL	AL
**FA-L-0629**	20		LI	LI	AL
**FA-L-0638**	15	10	LI	LI	AL
**FA-L-0644**	25		AL	LI	AL
**FA-L-0647**	29		LI	LI	AL
**FA-L-0650**	28		AL	AL	AL
**FA-L-0658**	16		LI	RO	AL
**FA-L-0660**	22		LI-PU?	LI-PU?	AL
**FA-L-0670**	39		LI	LI	AL
**FA-L-0672**	32		AL	LI	AL
**FA-L-0678**	25		LI	LI	AL
**FA-L-0682**	18		LI	LI	AL
**FA-L-0684**	18		LI	LI	AL
**FA-L-0756**	29		AL	PU	AL
**FA-L-0766**	21		LI	LI	AL
**FA-L-0771**	18		PU	LI	AL
**FA-L-0785**	31		AL	PU	AL
**FA-L-0797**	27		AL/LI	LI	RO/AL
**FA-L-0954**	25		LI	LI	AL
**FA-L-0955**	21		AL	PU	AL
**FA-L-0958**	31		LI	LI	AL
**FA-L-0972**	29		TR	TR	AL
**FA-L-0983**	18		PU	PU	AL
**FA-L-0995**	24		LI-PU?	LI-PU?	AL
**FA-L-0997**	15		AL	LI	AL
**FA-L-1006**	32*		RO	RO	AL
**FA-L-1007**	30		LI/AL	LI	AL
**FA-L-1008**	18		LI-PU?	LI-PU?	AL
**FA-L-1010**	33		PU	PU	AL
**FA-L-1011**	24		LI/AL	LI	AL
**FA-L-1012**	24		LI	LI	AL
**FA-L-1032**	29		LI	LI	AL
**FA-L-1040**	32		AL/LI	LI	AL
**FA-L-1299**	25		LI	LI	GO
**FA-L-781B**	20		AL	LI	AL
**FA-L-787B**	21		LI	LI	AL
**FV-6**			LI?	LI?	AL?
**G-1995ZC-L-506**	38		AL	LI	AL
**G-1995-ZC-L-589**	25		LI	LI	AL
**G-1995ZC-L-596**	27		IR	LI	AL
**G-2001MS-L-048**	40		LI	LI	AL
**G-2001MS-L-079**	14		AL	LI	RO/AL

**No. Inv L-**	**UTI.I.**	**UTI.D.**	**UTI.X.**	**MED.A.1**	**MED.A.2**
**FA-L-0599**	LI	LI	LI		
**FA-L-0600**	GO	LI	LI	96	15
**FA-L-0603**	RO	LI	LI		22
**FA-L-0613**	RO	PU	PU		
**FA-L-0629**	LI	LI	LI		
**FA-L-0638**	LI	LI	LI		
**FA-L-0644**	AL	LI	LI	86	28
**FA-L-0647**	RO	LI	LI		
**FA-L-0650**	AL	LI	LI	122	21
**FA-L-0658**	RO	LI	LI		
**FA-L-0660**	RO	LI-PU?	LI-PU?		
**FA-L-0670**	RO	LI	LI		
**FA-L-0672**	LI	LI	LI	157	27
**FA-L-0678**	RO	LI	RO		
**FA-L-0682**	RO	LI	LI		
**FA-L-0684**	RO	LI	LI		
**FA-L-0756**	GO	PU	PU		
**FA-L-0766**	LI	LI	LI		
**FA-L-0771**	RO	LI	LI		
**FA-L-0785**	PU	PU	PU	103	23
**FA-L-0797**	AL	AL/LI	LI	48	30
**FA-L-0954**	RO	LI	LI		
**FA-L-0955**	RO	PU	PU		19
**FA-L-0958**	GO?	LI	LI		
**FA-L-0972**	TR	LI	LI		
**FA-L-0983**	RO	LI	RO		
**FA-L-0995**	IR	LI	LI		
**FA-L-0997**	RO	LI	LI		11
**FA-L-1006**	RO	LI	RO		
**FA-L-1007**	LI	LI	LI	42	12
**FA-L-1008**	RO	LI-PU?	LI-PU?		
**FA-L-1010**	RO	PU	PU		
**FA-L-1011**	LI	LI	LI	34	22
**FA-L-1012**	LI	LI	LI		
**FA-L-1032**	LI	LI	LI	40	7
**FA-L-1040**	GO	GO	GO	54	31
**FA-L-1299**	AL	LI	LI		
**FA-L-781B**	GO	LI	LI	41	22
**FA-L-787B**	RO	LI	LI		
**FV-6**					
**G-1995ZC-L-506**	GA	LI	LI	185	45
**G-1995-ZC-L-589**	AL	LI	LI		
**G-1995ZC-L-596**	LI	LI	IR		
**G-2001MS-L-048**	AL	LI	LI		
**G-2001MS-L-079**	RO	AL	AL	18*	16*

**No. Inv L-**	**MED.R.1**	**MED.R.2**	**MED.S.1**	**MED.S.2**	**MED.I.1**
**FA-L-0599**			43	30	
**FA-L-0600**			27	10	
**FA-L-0603**			25	14	
**FA-L-0613**	40	26	26	13	
**FA-L-0629**			33	23	
**FA-L-0638**			37	22	
**FA-L-0644**			38	26	27
**FA-L-0647**			40	32	
**FA-L-0650**	99	22	27	25	30
**FA-L-0658**			38	18	
**FA-L-0660**			20	28	
**FA-L-0670**			26	27	
**FA-L-0672**			25	21	
**FA-L-0678**				30	
**FA-L-0682**			29	12	
**FA-L-0684**			28	15	
**FA-L-0756**			42	33	18
**FA-L-0766**			20	14	
**FA-L-0771**			28	18	
**FA-L-0785**					29
**FA-L-0797**					27
**FA-L-0954**			17	11	
**FA-L-0955**			21	14	
**FA-L-0958**			28	15	
**FA-L-0972**			18	18	
**FA-L-0983**					
**FA-L-0995**			40	13	
**FA-L-0997**			25	7	
**FA-L-1006**				20	
**FA-L-1007**			41	17	
**FA-L-1008**			25	9	
**FA-L-1010**			32	12	
**FA-L-1011**			26	11	
**FA-L-1012**			43	23	
**FA-L-1032**					
**FA-L-1040**			31	11	30
**FA-L-1299**			47	24	42
**FA-L-781B**			25	17	23
**FA-L-787B**			17	9	
**FV-6**					
**G-1995ZC-L-506**			35	28	23
**G-1995-ZC-L-589**			32	14	22
**G-1995ZC-L-596**			38	24	
**G-2001MS-L-048**			35	30	32
**G-2001MS-L-079**					

**No. Inv L-**	**MED.I.2**	**MED.D.1**	**MED.D.2**	**MED.X.1**	**MED.X.2**
**FA-L-0599**					
**FA-L-0600**					
**FA-L-0603**					
**FA-L-0613**					
**FA-L-0629**					
**FA-L-0638**					
**FA-L-0644**	10				
**FA-L-0647**					
**FA-L-0650**	23				
**FA-L-0658**					
**FA-L-0660**					
**FA-L-0670**					
**FA-L-0672**					
**FA-L-0678**					
**FA-L-0682**					
**FA-L-0684**					
**FA-L-0756**	16				
**FA-L-0766**					
**FA-L-0771**					
**FA-L-0785**	21				
**FA-L-0797**	24	42	10		
**FA-L-0954**					
**FA-L-0955**					
**FA-L-0958**					
**FA-L-0972**					
**FA-L-0983**					
**FA-L-0995**					
**FA-L-0997**					
**FA-L-1006**					
**FA-L-1007**					
**FA-L-1008**					
**FA-L-1010**					
**FA-L-1011**					
**FA-L-1012**					
**FA-L-1032**					
**FA-L-1040**	11	75	8	79	11
**FA-L-1299**	29				
**FA-L-781B**	12				
**FA-L-787B**					
**FV-6**					
**G-1995ZC-L-506**	18				
**G-1995-ZC-L-589**	8				
**G-1995ZC-L-596**					
**G-2001MS-L-048**	28				
**G-2001MS-L-079**		46*	9*	48*	8*

**No. Inv L-**	**UTI.ESP.**	**SIT.UTI.ESP.**	**L.ESP.**	**A.ESP.**	**P.ESP.**
**FA-L-0599**	TE	S	24	28	
**FA-L-0600**					
**FA-L-0603**					
**FA-L-0613**	TE	S	35	28	
**FA-L-0629**	TE	S	49	34	
**FA-L-0638**					
**FA-L-0644**	TE	S; I 29/11	26	29	
**FA-L-0647**	TE	S	40	32	
**FA-L-0650**	TE	S	25	24	
**FA-L-0658**					
**FA-L-0660**					
**FA-L-0670**					
**FA-L-0672**					
**FA-L-0678**					
**FA-L-0682**					
**FA-L-0684**					
**FA-L-0756**	TE				
**FA-L-0766**	TE	S			
**FA-L-0771**	TE	S			
**FA-L-0785**	TE	S			
**FA-L-0797**	TE	S;I			
**FA-L-0954**					
**FA-L-0955**					
**FA-L-0958**	TE	S			
**FA-L-0972**					
**FA-L-0983**	TE	S			
**FA-L-0995**	TE	S;I			
**FA-L-0997**	IN	A	4		
**FA-L-1006**	TE	S			
**FA-L-1007**	TE	S			
**FA-L-1008**	TE	S			
**FA-L-1010**	TE	S-A			
**FA-L-1011**	TE	S			
**FA-L-1012**	TE	S; R 40/10	49	35	
**FA-L-1032**	TE	S			
**FA-L-1040**	TE	S			
**FA-L-1299**					
**FA-L-781B**					
**FA-L-787B**					
**FV-6**					
**G-1995ZC-L-506**	TE	S	35	30	
**G-1995-ZC-L-589**	TE	S/I	66/23	34/25	
**G-1995ZC-L-596**	TE	S	38	24	
**G-2001MS-L-048**	TE	S/I	30/32	35/28	
**G-2001MS-L-079**	TE?	A			

**No. Inv L-**	**YACIM**	**No. Sondeo**	**ZON**	**Fase**	**Horiz.**	**ITEM**	**TIPO**	**MATERIA**
**G-2001MS-L-266**	Gatas		1/T1-N	III–IV		ALS	STA	ESQ
**G-S3-L-223**	Gatas				III	APE	STA	ESM
**G-S3-L-228**	Gatas					APE	STA	GNE
**G-S3-L-238**	Gatas					ALS	STA	PZA
**G-ZB-L-197**	Gatas					ALS	STA	MPS
**G-ZB-L-209**	Gatas					ALS	STA	PZA
**G-ZB-L-398**	Gatas				IVb	APE	STA	PZA
**G-ZB-L-400**	Gatas				IVa	APE	TA/CR	NESM
**G-ZC-L-086**	Gatas				IVc	ALS	STA	MPS
**G-ZC-L-114**	Gatas				IVb	ALS	STA	MPS
**G-ZC-L-163**	Gatas				Vb	ALS	STA	PZA
**G-ZC-L-190**	Gatas				Vb	APE	STA	PZA
**G-ZC-L-203**	Gatas				IVb	ALS	STA	ESM
**G-ZC-L-258**	Gatas				Vb	ALS	STA	MPS
**IF-12**	Ifre	Bibliogr				ALS	STA	PZA
**IF-13**	Ifre	Bibliogr				ALS	STA	PZA
**7042-7**	Murviedro					ALS	STA	ACA
**RI-1**	ncón de Almendri	c Bibliogr				ALS	STA	PZA
**TC-28**	Tres Cabezos	Bibliogr				ALS	STA	PZA
**TC-29**	Tres Cabezos	Bibliogr				ALS	STA	PZA
**TC-31**	Tres Cabezos	Bibliogr				ALS	STA	PZA
**TL-A1-7.1**	Tira del Lienzo		1	3		ALS	STA	ESQ
**TL-H10-5.1**	Tira del Lienzo		1	3		ALS	STA?	CAL
**TL-H10-67**	Tira del Lienzo		1	2		ALS	STA	MPS
**TL-H11-38**	Tira del Lienzo		1	2		ALS	STA	MPS

**No. Inv L-**	**PESO**	**CONS.**	**No. FR.**	**F.A.1**	**F.A.2**	**F.R.1**	**F.R.2**
**G-2001MS-L-266**	474	ENT	1	RT	RT	CX	CX
**G-S3-L-223**	256	ENT	1	RT	RT	CX	RT
**G-S3-L-228**	582	ENT	1	RT	CX	RT	RT
**G-S3-L-238**	48	ENT	1	RT	CX	CX	RT
**G-ZB-L-197**		FGS	1	RT	CX	CX	RT
**G-ZB-L-209**		FGS	1	RT	CX	CX	RT
**G-ZB-L-398**	220	ENT	1	RT	CX	CX	RT
**G-ZB-L-400**		ENT	1	RT	CX	CX	RT
**G-ZC-L-086**		FGT	1	RT	RT	CX	RT
**G-ZC-L-114**	50	ENT	1	RT	CX	CX	RT
**G-ZC-L-163**	590	ENT	1	RT	CX	CX	RT
**G-ZC-L-190**		FSM	1	RT	CX	CX	RT
**G-ZC-L-203**		FSM	1	RT	CX	CX	CV
**G-ZC-L-258**	215	ENT	1	RT	CX	CX	RT
**IF-12**							
**IF-13**							
**7042-7**	595	ENT	1	CX	CX	CV	CX
**RI-1**							
**TC-28**							
**TC-29**							
**TC-31**							
**TL-A1-7.1**	41*	FGT	2				
**TL-H10-5.1**	173	ENT	1	CX	CX	CX	CX
**TL-H10-67**	468	ENT	1	IR	CX	RT	CX
**TL-H11-38**	115*	FSM	1	RT	CX	RT	CX

**No. Inv L-**	**F.S.1**	**F.S.2**	**F.I.1**	**F.I.2**	**F.D.1**	**F.D.2**
**G-2001MS-L-266**	CX	CX	CX	CX	RT	CX
**G-S3-L-223**	CX	CX	CX	CX	RT	CX
**G-S3-L-228**	CX	CX	CX	CX	CX	CX
**G-S3-L-238**	CX	CX	CX	CX	RT	CX
**G-ZB-L-197**	CX	CX			RT	CX
**G-ZB-L-209**	CX	AG			RT	CX
**G-ZB-L-398**	CX	CX	CX	CX	RT	CX
**G-ZB-L-400**	CX	CX	CX	CX	RT	CX
**G-ZC-L-086**	CX	CX			CX	CX
**G-ZC-L-114**	CX	CX	CX	CX	RT	CX
**G-ZC-L-163**	CX	CX	CX	CX	RT	CX
**G-ZC-L-190**	CX	CX			RT	CX
**G-ZC-L-203**	CX	CX			RT	CX
**G-ZC-L-258**	CX	CX	CX	CX	RT	CX
**IF-12**						
**IF-13**						
**7042-7**	CX	CX	CX	CX	CX	CX
**RI-1**						
**TC-28**						
**TC-29**						
**TC-31**						
**TL-A1-7.1**						
**TL-H10-5.1**	CX	CX	CX	CX	CV	CX
**TL-H10-67**	CX	CX	CX	CX	RT	CX
**TL-H11-38**	CX	CX	RO	RO	RT	CX

**No. Inv L-**	**F.X.1**	**F.X.2**	**L.MAX.**	**A.MAX.**	**A.MIN.**
**G-2001MS-L-266**	CX	CX	150	56	
**G-S3-L-223**	RT	CX	131	41	
**G-S3-L-228**	RT	CX	173	62	
**G-S3-L-238**	CX	CX	91	24	
**G-ZB-L-197**	RT	CX		32	
**G-ZB-L-209**	RT	CX		25	
**G-ZB-L-398**	RT	CX	125	39	
**G-ZB-L-400**	RT	CX	151	59	
**G-ZC-L-086**					
**G-ZC-L-114**	RT	CX	82	28	
**G-ZC-L-163**	RT	CX	218	51	
**G-ZC-L-190**	RT	CX		29	
**G-ZC-L-203**	RT	CX		38	
**G-ZC-L-258**	RT	CX	152	46	
**IF-12**			120	26	
**IF-13**			132	24	
**7042-7**	CV	CX	188	58	
**RI-1**			177	32	
**TC-28**			182	36	
**TC-29**			172	22	
**TC-31**			194	44	
**TL-A1-7.1**					
**TL-H10-5.1**	CX	CX	115	34	
**TL-H10-67**	CX	CX	131	47	
**TL-H11-38**	RT	CX	80*	53*	

**No. Inv L-**	**G.MAX.**	**G.MIN.**	**UTI.A.**	**UTI.R.**	**UTI.S.**
**G-2001MS-L-266**	40		AL	LI	LI
**G-S3-L-223**	28		LI	LI	GO/AL
**G-S3-L-228**	40		LI	LI	GO
**G-S3-L-238**	15		LI	LI	AL
**G-ZB-L-197**	26		LI	LI	AL
**G-ZB-L-209**	15		LI	LI	AL
**G-ZB-L-398**	30		LI	LI	AL
**G-ZB-L-400**	31		RA/AL	LI	AL
**G-ZC-L-086**	15		PU	PU	AL
**G-ZC-L-114**	14		LI	LI	AL
**G-ZC-L-163**	33		LI	LI	AL
**G-ZC-L-190**	23		LI	LI	GO/AL
**G-ZC-L-203**	30		LI	LI	AL
**G-ZC-L-258**	17		PU	PU	AL
**IF-12**					AL?
**IF-13**					AL?
**7042-7**	40		LI	LI	AL
**RI-1**	25		LI		AL?
**TC-28**			LI		AL
**TC-29**			LI		AL
**TC-31**			LI		AL
**TL-A1-7.1**					
**TL-H10-5.1**	33		AL		AL
**TL-H10-67**	46		AL?	AL?	
**TL-H11-38**	22*				AL

**No. Inv L-**	**UTI.I.**	**UTI.D.**	**UTI.X.**	**MED.A.1**	**MED.A.2**
**G-2001MS-L-266**	AL	LI	LI	76	28
**G-S3-L-223**	GO	LI	LI		
**G-S3-L-228**	AL	LI	LI		
**G-S3-L-238**	AL	LI	LI		
**G-ZB-L-197**	RO	LI	LI		
**G-ZB-L-209**	RO	AL	LI		
**G-ZB-L-398**	GO	LI	LI		
**G-ZB-L-400**	GO	LI	LI	102	40
**G-ZC-L-086**	RO	RO	RO		
**G-ZC-L-114**	LI	LI	LI		
**G-ZC-L-163**	IR	LI	LI		
**G-ZC-L-190**	RO	LI	LI		
**G-ZC-L-203**	RO	LI	LI		
**G-ZC-L-258**	AL	LI	LI		
**IF-12**					
**IF-13**					
**7042-7**	LI	LI	LI		
**RI-1**					
**TC-28**					
**TC-29**					
**TC-31**					
**TL-A1-7.1**	AL				
**TL-H10-5.1**	AL			102	32
**TL-H10-67**	AL				
**TL-H11-38**					

**No. Inv L-**	**MED.R.1**	**MED.R.2**	**MED.S.1**	**MED.S.2**	**MED.I.1**
**G-2001MS-L-266**					32
**G-S3-L-223**			25	12	24
**G-S3-L-228**			60	34	28
**G-S3-L-238**			15	8	154
**G-ZB-L-197**			20	14	
**G-ZB-L-209**			15	8	
**G-ZB-L-398**			20	17	12
**G-ZB-L-400**			33	19	30
**G-ZC-L-086**					
**G-ZC-L-114**			17	8	
**G-ZC-L-163**			34	13	
**G-ZC-L-190**					
**G-ZC-L-203**					
**G-ZC-L-258**			28	25	30
**IF-12**					
**IF-13**					
**7042-7**					
**RI-1**					
**TC-28**					
**TC-29**					
**TC-31**					
**TL-A1-7.1**					
**TL-H10-5.1**			19	8	26
**TL-H10-67**					42
**TL-H11-38**			31	20	

**No. Inv L-**	**MED.I.2**	**MED.D.1**	**MED.D.2**	**MED.X.1**	**MED.X.2**
**G-2001MS-L-266**	34				
**G-S3-L-223**	18				
**G-S3-L-228**	23				
**G-S3-L-238**	9				
**G-ZB-L-197**					
**G-ZB-L-209**					
**G-ZB-L-398**	15				
**G-ZB-L-400**	14				
**G-ZC-L-086**					
**G-ZC-L-114**					
**G-ZC-L-163**					
**G-ZC-L-190**					
**G-ZC-L-203**					
**G-ZC-L-258**	23				
**IF-12**					
**IF-13**					
**7042-7**					
**RI-1**					
**TC-28**					
**TC-29**					
**TC-31**					
**TL-A1-7.1**					
**TL-H10-5.1**	13				
**TL-H10-67**	36				
**TL-H11-38**					

**No. Inv L-**	**UTI.ESP.**	**SIT.UTI.ESP.**	**L.ESP.**	**A.ESP.**	**P.ESP.**
**G-2001MS-L-266**	TE	I	32	34	
**G-S3-L-223**					
**G-S3-L-228**	TE	CO			
**G-S3-L-238**					
**G-ZB-L-197**					
**G-ZB-L-209**					
**G-ZB-L-398**	TE	TE			
**G-ZB-L-400**	TE	SA;A41/18			
**G-ZC-L-086**					
**G-ZC-L-114**					
**G-ZC-L-163**					
**G-ZC-L-190**					
**G-ZC-L-203**	TE	TE			
**G-ZC-L-258**	TE	TE			
**IF-12**					
**IF-13**					
**7042-7**	TE	S	40	32	
**RI-1**	TE	S			
**TC-28**					
**TC-29**					
**TC-31**					
**TL-A1-7.1**					
**TL-H10-5.1**					
**TL-H10-67**	TE	I	42	36	
**TL-H11-38**	TE	S	31-20		
