# Supplementary material for: Dataset on the evidence of bee products processing: A functional definition of a specialized type of macro-lithic tool
Source: Data Brief. 2017 Sep 9;14:738–58. doi: 10.1016/j.dib.2017.08.044 (PMC5602746; doi:10.1016/j.dib.2017.08.044)
Supplement: Supplementary file 1 — Supplementary material [file mmc1.docx]

*Data article*

**Title:** Dataset on the evidence of bee products processing: a functional definition of a specialized type of macro-lithic tool

Conflict of interest form^[[1]](#footnote-1)^

**Authors:** Mireia Ache^a^, Selina Delgado-Raack ^a,*^, Elena Molina^a,b^, Roberto Risch ^a^, Antoni Rosell-Melé^b,c^

**(*) Corresponding author email:** [selina.delgado@uab.cat](mailto:Selina.delgado@uab.cat) (Selina Delgado-Raack, corresponding author)

I, Selina Delgado-Raack, as corresponding author and acting on behalf of the rest of the co-authors of the aforementioned article, wish to confirm that there are no known conflicts of interest associated with this publication and there has been no significant financial support for this work that could have influenced its outcome.

I confirm that the manuscript has been read and approved by all named authors and that there are no other persons who satisfied the criteria for authorship but are not listed.

I further confirm that the order of authors listed in the manuscript has been approved by all of us.

I confirm that I have given due consideration to the protection of intellectual property associated with this work and that there are no impediments to publication, including the timing of publication, with respect to intellectual property. In so doing I confirm that I have followed the regulations of our institutions concerning intellectual property.

I understand that, as Corresponding Author, I am the sole contact for the Editorial process (including Editorial Manager and direct communications with the office). I am responsible for communicating with the other authors about progress, submissions of revisions and final approval of proofs.


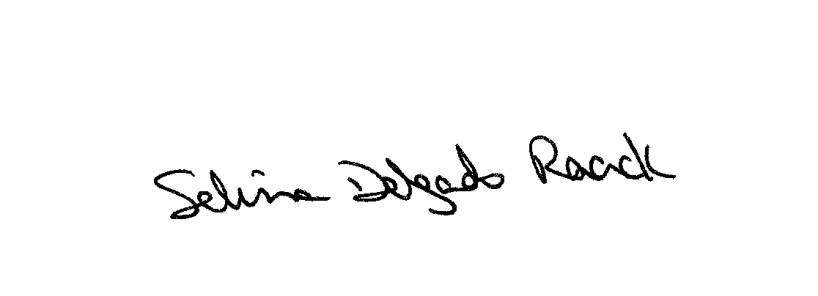


1. We have included this ad-hoc word since the link in the website to the “Conflict of Interest” form is broken. (See web paragraph “*Conflict of Interest is mandatory for revision, so while submitting please submit the file by selectin the description from the drop down. Please click here to download the Conflict of Interest form*”). [↑](#footnote-ref-1)
